# Pleural Fluid Cytology of the Polymorphous Variant of EBV-Positive Diffuse Large B-Cell Lymphoma: First Report and Distinction from a Reactive Process

**DOI:** 10.1155/2013/459279

**Published:** 2013-09-03

**Authors:** Lisa Rooper, Christopher D. Gocke, Deborah A. Belchis

**Affiliations:** ^1^Department of Pathology, Johns Hopkins University, Baltimore, MD 21287, USA; ^2^Johns Hopkins Bayview Medical Center, Pathology 1 Floor, Room AA158, 4940 Eastern Avenue, Baltimore, MD 21224, USA

## Abstract

EBV-positive diffuse large B-cell lymphoma of the elderly is a newly described aggressive lymphoma predominantly affecting patients >50 years of age. Patients may present with nodal and/or extranodal involvement. The lung is one of the more common extranodal sites. The incidence of pleural fluid involvement is less well described. In one study by Oyama et al., pleural effusions were noted in nine percent of cases. Identification of pleural fluid involvement could be important as it may carry prognostic importance in staging (it typically occurs more often in cases with widespread disease), and it could be a relatively easy means of establishing a diagnosis in newly presenting cases. We report the first description of the pleural fluid cytology in a case of EBV-positive diffuse large B-cell lymphoma of the elderly.

## 1. Introduction

EBV-positive diffuse large cell lymphoma (EBV+ DLBCL) of the elderly is a newly recognized aggressive B-cell lymphoproliferative disorder. Initially characterized by Oyama et al. in 2003 [1], this entity was provisionally recognized as a subtype of DLBCL in the WHO classification in 2008 [2]. Unlike other EBV-associated lymphomas, EBV+ DLBCL of the elderly occurs exclusively in patients with no history of immunodeficiency. As the name suggests, these patients are older and have a median age of 71 years, with 20%–25% of cases occurring in patients older than 90. Rare cases have been described in younger patients. Patients may present with nodal or extranodal disease. While pleural effusions have been noted in these patients, the cytologic features have not been described. Identification of pleural fluid involvement may provide a rapid diagnosis as well as assist in staging the tumor. This is the first description of the cytologic features of pleural fluid involved by EBV+ DLBCL of the elderly.

## 2. Case Report

A 64-year-old man presented to an outside hospital with fatigue, fever, chills, nights sweats, and a 65 pound weight loss over a 2.5 month period. A CT scan demonstrated hilar and mediastinal lymphadenopathy and splenomegaly. An extensive workup was performed including lymph node and skin biopsies and cytologic evaluation of pleural effusion. All sites showed a polymorphic population of T and B-cells with occasional rare atypical CD20 positive B-cells. IgH and kappa and lambda studies on the skin biopsy failed to identify a clonal population. While the findings were worrisome for lymphoma, a definitive diagnosis could not be made. The decision was made to transfer the patient to our institution.

**Figure 1:**
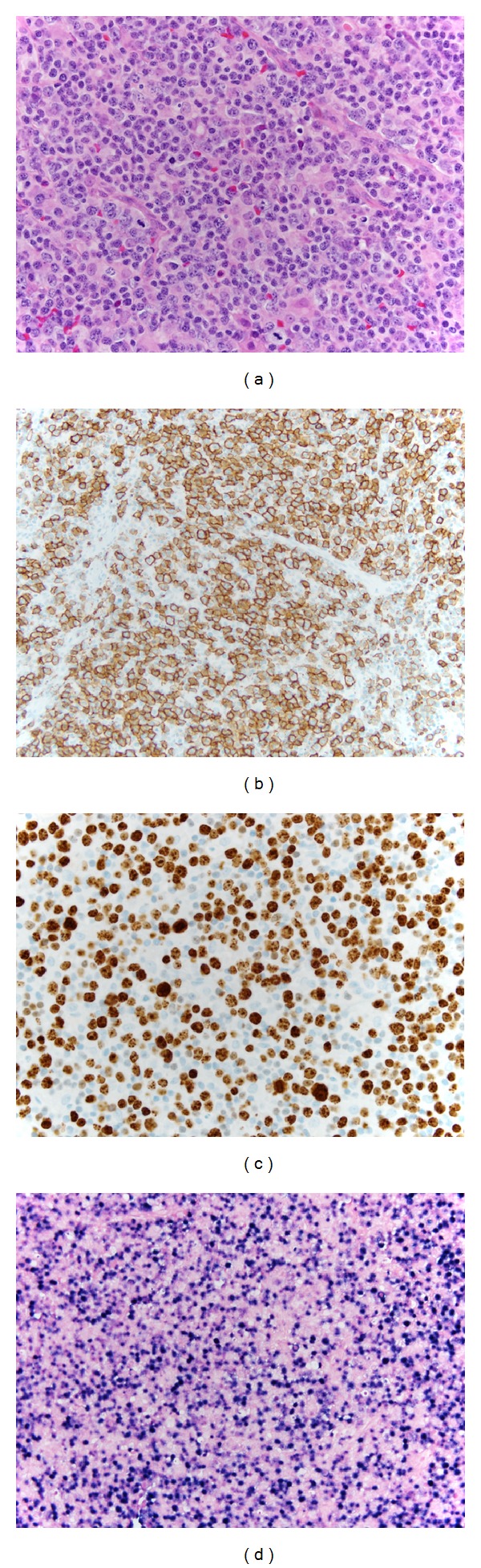
(a) Lymph node with small and large atypical lymphoid cells admixed with histiocytes, immunoblasts, plasma cells and eosinophils; H&E 400x. (b) CD20 immunostain highlighting the small and large atypical B-cells. (c) Ki-67 highlighting the high proliferative rate. (d) EBER-ISH showing diffuse positivity.

On admission to our hospital, the outside histopathology was reviewed and the diagnoses confirmed. Given the strong clinical suspicion of lymphoma, a second lymph node was biopsied. The lymph node contained a polymorphous population of medium-to-large-sized lymphocytes with clumped chromatin, multiple nucleoli, a scant-to-moderate amount of cytoplasm, and frequent mitotic figures (Figure 1(a)). There were also multiple intermixed T lymphocytes, immunoblasts, histiocytes, eosinophils, and plasma cells. This infiltrate focally extended into surrounding fibroadipose tissue. A panel of immunostains identified the atypical lymphocytes as strongly CD20 positive (Figure 1(b)) and Bcl-2, CD-10, and Bcl-6 negative. The Ki-67 proliferation index in these cells was high (Figure 1(c)). EBV-encoded RNA in situ hybridization (EBER-ISH) was positive in the large atypical cells (Figure 1(d)). The morphological features and immunophenotype, when taken in the clinical context, were consistent with Epstein Barr virus positive diffuse large B-cell lymphoma (EBV+ DLBCL) of the elderly.

**Figure 2:**
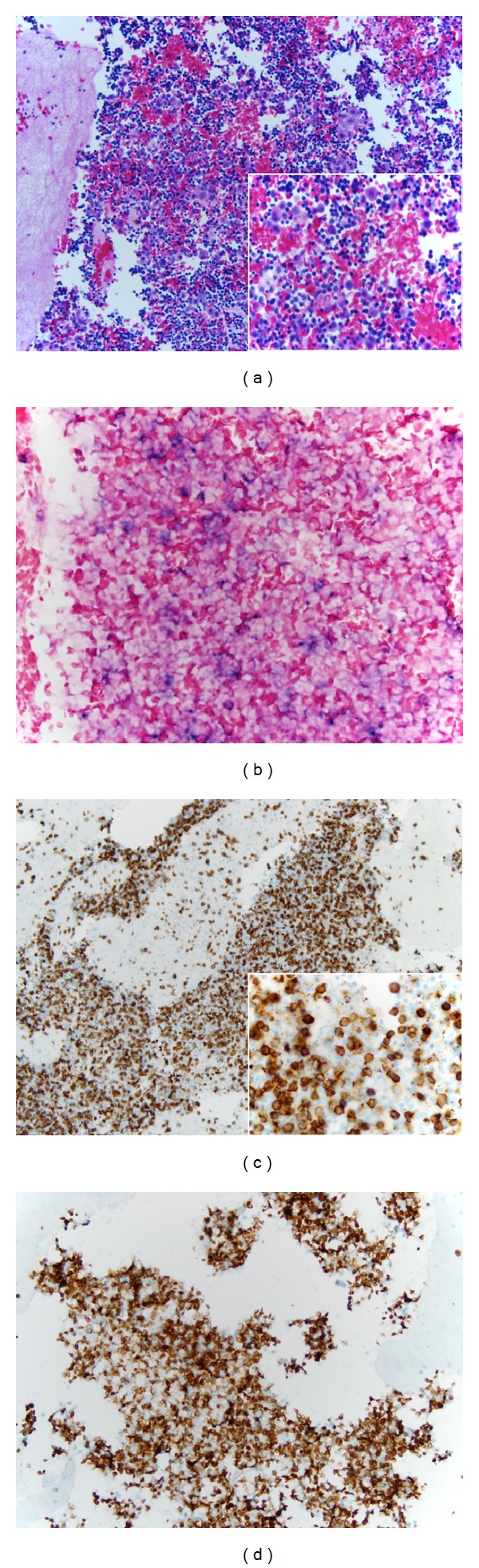
(a) Pleural fluid cell block showing mixed population with background atypical cells H&E 200x. Inset highlighting the hidden atypical cells H&E 400x. (b) EBER with positive atypical cells. (c) and (d) CD20 and CD3 stains 100x, respectively, showing a mixed population with the atypical cells positive for CD20 (inset 600x).

The patient started therapy and developed sepsis complicated by worsening dyspnea. A pleural effusion and pulmonary infiltrate were noted. Pneumonia was suspected clinically, and a thoracentesis was performed. Cytologic and immunohistochemical evaluation of the fluid demonstrated a polymorphous population of B and T cells (Figures 2(a), 2(b), and 2(c)). Hidden in this population were rare atypical large cells positive for CD20 (Figure 2(b)). EBER-ISH demonstrated positivity in virtually all of the larger atypical cells, confirming involvement of the pleural fluid by the patient's lymphoma (Figure 2(d)). An HHV-8 immunostain was negative. The patient passed away a few days later due to complications of chemotherapy. 

## 3. Discussion

EBV+ DLBCL of the elderly is a relatively newly described entity with an aggressive course. By definition, it arises predominantly in immunocompetent adults older than 50 years of age, although younger patients have been described [3, 4]. Patients may present with nodal or extranodal disease or both. In a study of 96 cases by Oyama et al., in 20% of cases the disease was limited to extranodal sites, in 31% only nodal involvement was noted, and in 49% both nodal and extranodal disease were present [5]. The skin was the most common site of extranodal involvement. A pleural effusion was noted in 9% of cases. Morphologically, EBV+ DLBCL of the elderly is separated into two patterns: a polymorphous subtype consisting of atypical lymphoid cells admixed with varying numbers of T cells, plasma cells, and histiocytes and a monomorphic subtype characterized by sheets of large, atypical transformed B-cells. Recognition of the latter as a malignant process is relatively straightforward. The polymorphic subtype can be more problematic depending on the proportion of atypical cells present and the clinical index of suspicion. There is increasing consensus that the subtypes are extremes on a continuum and are not of clinical importance.

Since its initial recognition, the morphologic overlap between EBV+ DLBCL of the elderly and reactive EBV lymphoproliferative disorders, classic Hodgkin lymphoma (cHL), and T cell-rich B-cell lymphoma has been recognized [6–8]. Clinically, EBV+ cHL and age-related EBV+ lymphoproliferative disorders both tend to occur in the elderly. Immunohistochemistry has been suggested as a way to separate these 2 entities with the tumor cells of EBV+ DLBCL of the elderly typically CD20 positive with variable staining for CD15 and the tumor cells of cHL typically CD15 positive with variable staining for CD20. Light chain restriction may be difficult to demonstrate [2]. If it is present, demonstration of monoclonality can help separate EBV+ DLBCL of the elderly from a reactive process. The presence of a pleural effusion does not help to refine the diagnosis as many lymphomas can be associated with pleural effusion, usually arising as a part of a systemic process, with the incidence varying from 6% to 20% in Hodgkin and non-Hodgkin lymphomas [9]. Certain lymphomas almost exclusively involve serous cavities, most notably primary effusion lymphoma, plasmablastic lymphoma, and DLBCL of chronic inflammation [8, 10, 11]. However, even in other types of lymphomas, the pleural cavity may be the sole site of involvement. Two cases of PTLD presenting as a pleural effusion without disease elsewhere are described and in both cases recognizing the process as PTLD was challenging and yet essential for appropriate treatment [12, 13]. Even in our case where the diagnosis of EBV+ DLBCL was known, the cytology was challenging, requiring special stains. Therefore, the differential diagnosis is broad. Primary effusion lymphoma can be separated from EBV+ DLBCL by HHV-8 positivity. Plasmablastic lymphoma and PTLD are EBV driven lymphomas, occurring in immunocompromised hosts as opposed to the presumptively immunocompetent setting of EBV+ DLBCL of the elderly. Recognition of pleural involvement in lymphoma is important as it is associated with a more aggressive course [14, 15].

In summary, we report the first description of the cytologic findings of EBV+ diffuse large B-cell lymphoma involving the pleural fluid. Diagnosis of lymphomas by cytologic examination of fluid is well recognized and accepted. However, pitfalls remain due to cytologic overlap among lymphoma subtypes. EBV+ DLBCL of the elderly has just recently been added to WHO classification of diffuse large B-cell lymphomas. In at least a subset of cases, pleural effusions are present either at the time of presentation or subsequently. Pleural fluid analysis may be a comparatively easy target for initial diagnosis. The polymorphous infiltrate, common to other EBV driven processes, can be misleading and lead to a diagnosis of a reactive process. A high index of suspicion is important to make the diagnosis. Therefore, this case also demonstrates the importance of adding EBV in situ hybridization to the list of ancillary studies performed in the evaluation of polymorphic pleural effusion cytology specimens. In our case, the initial review revealed rare, partially obscured atypical lymphoid cells in a polymorphic background, initially interpreted as being reactive. Clinically, the patient was believed to have therapy-induced pneumonia, and the cytology supported this. Cytomorphology alone was not diagnostic. The correct diagnosis required EBER-ISH with careful clinical correlation.
